# ZRSR2 loss causes aberrant splicing in JAK2^V617F^‐driven myeloproliferative neoplasm but is not sufficient to drive disease progression

**DOI:** 10.1002/hem3.70225

**Published:** 2025-09-16

**Authors:** Ranran Zhang, Jasmin Straube, Yashaswini Janardhanan, Rohit Haldar, Leanne Cooper, Noah Hayes, Charles Laurore, Chulwoo J. Kim, Marlise R. Luskin, Robert C. Lindsley, Maximilian Stahl, Ann Mullally, Anna E. Marneth, Megan Bywater, Steven W. Lane

**Affiliations:** ^1^ Cancer Research Program QIMR Berghofer Medical Research Institute Herston Queensland Australia; ^2^ Faculty of Health, Medicine and Behavioural Sciences The University of Queensland Brisbane Queensland Australia; ^3^ Department of Medicine Brigham and Women's Hospital, Harvard Medical School, Division of Hematology Boston Massachusetts USA; ^4^ Department of Medical Oncology Dana‐Farber Institute Boston Massachusetts USA; ^5^ Department of Medicine Stanford University School of Medicine, Division of Hematology Stanford California USA; ^6^ Hematology Division VA Palo Alto Health Care System Palo Alto California USA; ^7^ Laboratory of Hematology, Department of Laboratory Medicine Radboud University Medical Center, Radboud Institute for Medical Innovation Nijmegen The Netherlands; ^8^ Cancer Care Services Royal Brisbane and Women's Hospital Herston Queensland Australia

Myeloproliferative neoplasms (MPNs), including polycythemia vera (PV), essential thrombocythemia (ET), and primary myelofibrosis (PMF), are clonal disorders driven by mutations in hematopoietic stem cells (HSCs).^1^, ^2^ *JAK2* ^V617F^ is the most recurrent driver mutation in MPN and results in the constitutive activation of the JAK‐STAT pathway.^3^, ^4^, ^5^, ^6^ Mutations in *ZRSR2* have been found in *JAK2* ^V617F^‐driven MPNs and are associated with disease progression and poor prognosis. However, the functional consequences of mutations in *ZRSR2* in terms of disease progression in *JAK2* ^V617F^‐driven MPN have not been determined. In MPN, somatic mutations in *ZRSR2* occur across the entire coding transcript, are predicted to confer a loss‐of‐function, and are primarily observed in male patients (Supporting Information: Figure S1).^7^, ^8^, ^9^ Herein, using CRISPR‐Cas9, we generated concomitant ZRSR2 loss in JAK2‐mutated human megakaryoblastic cell lines (SET2, CHRF‐288‐11) and a mouse model of MPN to investigate its role in promoting disease progression. We confirmed ZRSR2 loss in both human cell lines and this mouse model by sequencing and immunoblotting (Supporting Information: Figures S2 and S3).

*ZRSR2* is mainly involved in minor spliceosome assembly, with *ZRSR2* loss previously shown to result in the mis‐splicing of U12‐type introns in myelodysplastic syndromes (MDSs).^10^, ^11^ We therefore performed replicate multivariate analysis of transcript splicing (rMATS)^12^ to detect differential intron retention between *ZRSR2* knockout (KO) megakaryoblastic cells and controls, and identified significantly increased retained intron (RI) events false discovery rate [FDR] < .05) in SET‐2 *ZRSR2* KO cell lines, but not in CHRF‐288‐11. However, in both megakaryoblastic cell lines, RI events tended to be more prominent in genes containing U12‐type introns in the context of *ZRSR2* loss (Figure 1A). Differential gene expression analysis was performed to assess how *ZRSR2* loss impacts transcription. Gene set enrichment analysis (GSEA) showed enrichment for genes containing U12‐type introns in both ZRSR2 KO megakaryoblastic cell lines versus control cells (Supporting Information: Figure S4A). We observed significant enrichment for megakaryocyte progenitors (MkPs) and E2F target gene sets, which are involved in MkPs and myeloid differentiation, in CHRF‐288‐11 *ZRSR2* KO cells, but not in SET‐2 *ZRSR2* KO cells (Supporting Information: Figure S4B). Taken together, we conclude that *ZRSR2* loss causes U12‐type intron retention and transcriptional changes in genes containing U12‐type introns in human *JAK2*‐mutant megakaryoblastic cell lines.

**Figure 1:**
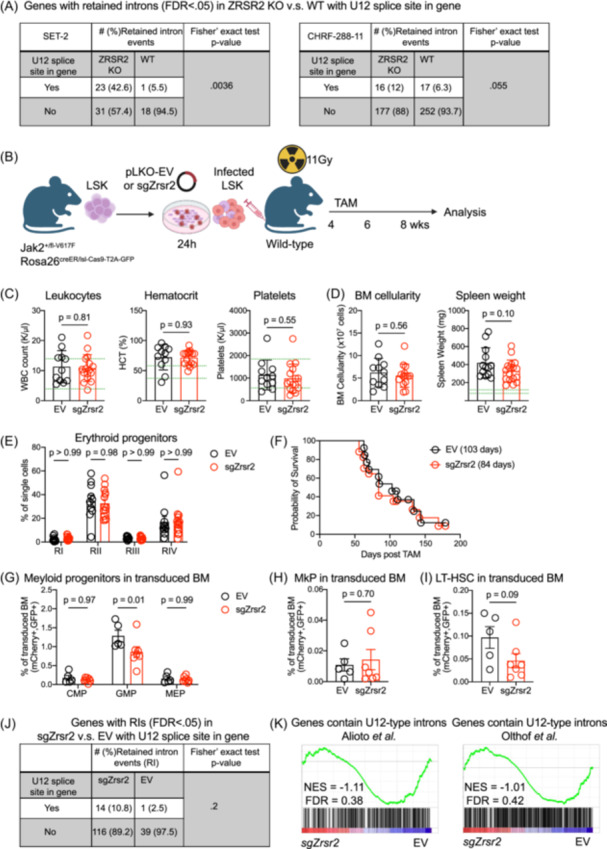
**ZRSR2 loss does not accelerate *Jak2* ^V617F^‐driven MPN in vivo. (A)** Replicate multivariate analysis of transcript splicing (rMATS)^12^ retained introns (RIs) in human megakaryoblastic cell lines. RI events are grouped by the presence or absence of a U12 splice site in the gene. Fisher's exact test, P = 0.0036, in SET‐2 cells and Fisher's exact test, P = 0.055, in CHRF‐288‐11 cells. **(B)** Experimental setup of *Zrsr2* loss in *Jak2* ^V617F^ mice. **(C)** Peripheral blood parameters in mice transplanted with *Jak2* ^V617F^ cells expressing sgZrsr2 (sgZrsr2) or empty vector (EV) control: leukocyte number, hematocrit, and platelet number. **(D)** Bone marrow cellularity and spleen weights in mice transplanted with *Jak2* ^V617F^ cells expressing sgZrsr2 or control vector. **(E)** Percentages of erythroid progenitors in spleen. CD71^high^/Ter119^low^, CD71^high^/Ter119^high^, CD71^dim^/Ter119^high^, and CD71^−^/Ter119^high^ were defined as Region I (RI), Region II (RII), Region III (RIII), and Region IV (RIV), respectively. In erythropoiesis, these regions are enriched in Pro, Baso, Poly, and Ortho erythroblasts, respectively. **(F)** Kaplan–Meier survival curve of mice transplanted with *Jak2* ^V617F^ cells expressing sgZrsr2 or control vector. **(G–I)** Percentages of hematopoietic stem and progenitor cells (HSPCs) compartment in the bone marrow: common myeloid progenitor (CMP), granulocyte/macrophage progenitor (GMP), and megakaryocytic/erythroid progenitor (MEP) (G), megakaryocytic progenitor (MkP) (H), and long‐term hematopoietic stem cells (LT‐HSCs) (I). Murine HSPC compartment subsets were defined as CMP (Lineage^−^Sca‐1^−^cKit^+^CD34^+^FcγR^−^), GMP (Lineage^−^Sca‐1^−^cKit^+^CD34^+^FcγR^+^), MEP (Lineage^−^Sca‐1^−^cKit^+^CD34^−^FcγR^−^), MkP (Lineage^−^Sca‐1^−^cKit^+^CD150^+^CD41^+^), and LT‐HSCs (Lineage^−^Sca‐1^+^cKit^+^CD150^+^CD48^−^). Lineage markers include CD3e, B220, Ter‐119, Mac‐1, Gr‐1, and CD5. Green dotted lines show physiological data in C57BL/6J mice (www.jax.org/phenome). Data were collected as the mice became moribund and were pooled from three independent experiments (*n* = 2–5 mice/genotype/experiment). Results are presented as mean ± s.e.m. Each circle represents one mouse, and P‐values are indicated. **(J)** rMATS identified RIs FDR < 0.05) grouped by the presence or absence of a U12 splice site in the gene, Fisher's exact test, P = 0.2. **(K)** Gene set enrichment analysis (GSEA) on gene sets containing U12‐type introns comparing transduced Lineage^−^Sca‐1^+^c‐Kit^+^ (LSK) cells from *Jak2* ^V617F^‐sgZrsr2 and *Jak2* ^V617F^‐EV recipient mice. BM, bone marrow; FDR, false discovery rate; HCT, hematocrit; KO, knockout; NEM, normalized enrichment score; TAM, tamoxifen; WBC, white blood cell; WT, wild type.

*Zrsr2* loss in *Jak2* ^V617F^‐driven murine MPN was achieved by isolating bone marrow (BM) Lineage^−^Sca‐1^+^c‐Kit^+^ (LSK) cells from male donor mice (*Jak2* ^*+/*fl‐V617F^, *Rosa26* ^creER/lsl‐Cas9‐T2A‐GFP^), transducing them with a lentiviral vector expressing sgRNA targeting *Zrsr2* (sgZrsr2), and transplanting into irradiated wild‐type recipient mice. After 4 weeks of engraftment, mice were fed Tamoxifen‐chow for 2 weeks to induce CreER and *Zrsr2* editing (Figure 1B). *Zrsr2*‐edited recipients and unedited controls all developed a lethal MPN resembling human PV with elevated hematocrit, splenomegaly, and expansion of erythroid precursors (Figure 1C–E). The disease phenotype observed was not associated with the sex of *Zrsr2*‐edited recipients (Supporting Information: Figure S5). No significant differences in peripheral MPN disease parameters, main hematopoietic lineages, or survival were observed with *Zrsr2* loss, except for an increase in peripheral progenitor cells (cKit^+^) (Figure 1C–F, Supporting Information: Figure S6A–D). *ZRSR2* mutations have been described to occur frequently in PMF, a disease state characterized by abnormal megakaryocyte development.^13^ However, except for a slight decrease in granulocyte/macrophage progenitors (GMPs) in *Jak2* ^V617F^‐sgZrsr2 mice, we did not observe any difference in the proportion of megakaryocyte/erythroid progenitors (MEPs), common myeloid progenitors (CMP), MkPs, or long‐term HSCs (LT‐HSCs) in the transduced donor cell population across conditions (Figure 1G–I). Furthermore, no difference was detected in the frequency of transduced donor cells within these populations (Supporting Information: Figure S6E), demonstrating that *Zrsr2* disruption does not confer a competitive advantage in combination with *Jak2* ^V617F^ within the HSPC compartment. Histopathological analysis across both conditions was consistent with a PV phenotype, showing effacement of splenic architecture with erythroid and megakaryocytic hyperplasia and the megakaryocytes in BM displayed atypical nuclear features and clustering, with no evidence of disease progression observed with *Zrsr2* loss (Supporting Information: Figure S6F).

We further investigated whether *Zrsr2* disruption affected intron retention in BM LSK cells and identified a higher number of RIs in *Jak2* ^V617F^‐sgZrsr2 mice. However, these RIs were less restricted by type, with fewer occurring in genes containing the U12 splice site in mouse LSK cells compared to human *ZRSR2* KO megakaryoblastic cells (Figure 1A,J). Furthermore, we identified only four genes with RIs regulated by *ZRSR2* in both species (Supporting Information: Figure S7A), indicating that *Zrsr2* loss results in different levels of mis‐splicing of genes containing U12‐type introns between cell types and/or between humans and mice, with notably less predilection in murine cells. Consistent with this, the bulk gene expression profiles of murine genes containing U12‐type introns, in contrast to human cells, did not demonstrate significant enrichment of transcripts containing U12‐type introns in either direction (Figure 1K). However, consistent with genetic profiles of chronic‐stage MPN patients that progress to MF who show megakaryocytic hyperplasia and aberrant mobilization of HSCs, we observed significant enrichment for MkP‐associated gene sets and transcriptional changes associated with genes upregulated in HSCs in *Jak2* ^V617F^‐sgZrsr2 compared to *Jak2* ^V617F^‐EV cells (Supporting Information: Figure S7B). Taken together, *Zrsr2* loss causes modest intron retention and minor transcriptional changes in murine LSK cells. However, the transcriptional changes that occur as a consequence of *Zrsr2* loss are not sufficient to induce disease progression of *Jak2* ^V617F^‐driven murine MPN in vivo.

Our results are similar to the report of Madan et al., in that *Zrsr2* deficiency also led to fewer U12‐type mis‐splicing events observed in murine hematopoietic cells compared to human MDS or leukemia cell lines.^14^ In this study, they raised the issue that *Zrsr1*, a homolog of *Zrsr2* expressed in mice but not in humans, may compensate for the impact of *Zrsr2* loss in regulating RNA splicing in mice. *Zrsr1* expression is not affected by *Zrsr2* KO in our murine model (Supporting Information: Figure S7C). The compensatory role of *Zrsr1* for *Zrsr2* in hematopoietic development in mice may explain the attenuated intron retention and milder disease phenotype observed in our murine model compared to human cells. However, the frequent co‐occurrence of mutations in epigenetic regulators and splicing regulators in MPN samples harboring a *ZRSR2* mutation may also suggest that a cooperative role for these molecular lesions with ZRSR2 is required for the progression of MPN.^15^, ^16^, ^17^, ^18^

From a published MPN cohort containing 12 patients with concurrent *ZRSR2* mutations in 1289 *JAK2* ^V617F^ patients,^7^ we found that *ZRSR2* mutations were significantly more frequent in *JAK2* ^V617F^ cases with MF compared to those with PV and ET (P < 0.0001, Pearson's chi‐squared test), with frequencies of 3.69%, 0.28%, and 0.41%, respectively. Among the patients with co‐mutations in *JAK2* ^V617F^ and *ZRSR2*, eight cases had one or more additional mutations, most frequently detected in the epigenetic regulators *ASXL1* and *TET2* (Figure 2A). It is noteworthy that these co‐occurring mutations were more commonly associated with MF rather than ET or PV, including chromatin modifiers *ASXL1* and *EZH2*, or DNA methylation regulator *TET2* (Figure 2A). Moreover, the frequency of *ASXL1, EZH2*, and *TET2* mutations was significantly higher in cases co‐mutated with *ZRSR2* compared to those without *ZRSR2* mutations (*ASXL1*: 33.3% vs. 6.81%, P = 0.0074; *EZH2*: 16.67% vs. 1.72%, P = 0.019; *TET2*: 33.3% vs. 14.41%, P = 0.0839; Fisher's exact test). These data suggest that high‐risk mutations in chromatin modifiers *ASXL1* and *EZH2* that predispose to progression of MPN to MF are more frequently present in *JAK2* ^V617F^ MPN patients with *ZRSR2* mutations. We validated these findings in an independent data set that included 18 *ZRSR2*‐mutated MPN patients within a cohort of 990 MPN patients seen at DFCI (Supporting Information: Table S1). Of these *JAK2* and *ZRSR2* co‐mutated patients, we observed similar findings, with eight patients having at least one additional mutation, occurring most frequently in *ASXL1* (46.2%) and *TET2* (30.8%), respectively (Figure 2B). Interestingly, two patients lost *ZRSR2* mutations upon progression to SMF, and three patients acquired their *ZRSR2* mutation in a late disease stage, after onset of MF (Figure 2C), suggesting that *ZRSR2* mutations are not primary drivers of myelofibrosis progression. Using the DFCI cohort, we further explored the relationship between the *ZRSR2* mutations and the MPN phenotype by analyzing the correlation matrix of ZRSR2 variant allele frequency (VAF) with blood parameters. Consistent with clinical observations, patients with MF or SMF showed a trend of higher ZRSR2 VAF and elevated platelet counts, as well as a trend of lower hemoglobin levels and white blood cell counts (Supporting Information: Figure S8). Together, using two independent retrospective data sets, we identified that *ZRSR2* mutations are enriched in patients with MF and are typically found in genetically complex MPN. These findings suggest that *ZRSR2* mutations alone are insufficient to drive fibrotic progression of MPN. Consequently, functional and mechanistic studies to determine whether *ZRSR2* mutations predispose to genetic instability and how they cooperate with epigenetic regulators to promote disease progression in MPN are planned.

**Figure 2:**
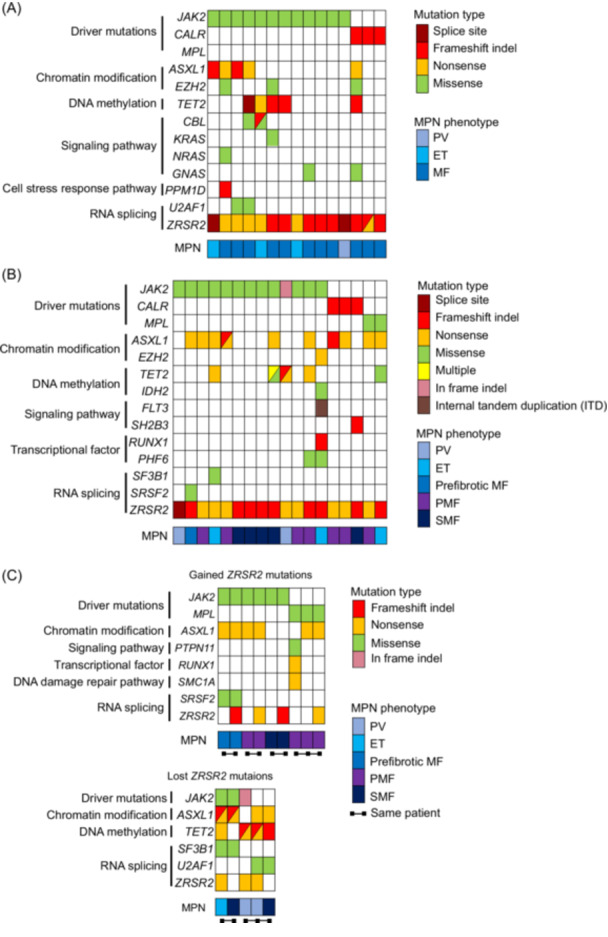
**Co‐occurring high‐risk mutations correlate with disease progression in *JAK2* ^V617F^‐driven myeloproliferative neoplasm (MPN). (A, B)** Heatmaps of somatic mutations in *ZRSR2*‐mutated MPN patients from a published cohort^7^ (*n* = 15 patients) (A) and from the Dana‐Farber Cancer Institute (DFCI) cohort (*n* = 18 patients) (B). **(C)** Heatmap of somatic mutations in sequential samples from MPN patients carrying a *ZRSR2* mutation on at least one time point. Data are from the DFCI. Connected dots indicate that samples are from the same patient. In both cohorts, somatic mutation types include splice site, frameshift indel, in‐frame indel, nonsense, missense, and multiple internal tandem duplication (ITD). Somatic mutations are categorized by genes involved in driving the development of an MPN phenotype (driver mutations), chromatin modification, DNA methylation, signaling pathway, transcription regulation, DNA damage repair pathway, cell stress response pathway, and RNA splicing. MPN patient phenotypes include polycythemia vera (PV), essential thrombocythemia (ET), myelofibrosis (MF), primary myelofibrosis (PMF), prefibrotic myelofibrosis (prefibrotic MF), and secondary myelofibrosis (SMF).

In summary, we determine that ZRSR2 loss using CRISPR‐Cas9 gene editing causes minor changes in intron retention and transcription; however, this is not sufficient to induce disease progression in preclinical models of JAK2^V617F^‐driven MPN.

## AUTHOR CONTRIBUTIONS

**Ranran Zhang**: Conceptualization; methodology; data curation; investigation; formal analysis; writing—original draft; visualization; writing—review and editing; validation. **Jasmin Straube**: Writing—review and editing; formal analysis; data curation; methodology; software; validation. **Yashaswini Janardhanan**: Investigation. **Rohit Haldar**: Investigation. **Leanne Cooper**: Investigation. **Noah Hayes**: Investigation. **Charles Laurore**: Investigation. **Chulwoo J. Kim**: Formal analysis. **Marlise R. Luskin**: Resources. **Robert C. Lindsley**: Resources. **Maximilian Stahl**: Resources. **Ann Mullally**: Conceptualization; writing—review and editing; funding acquisition; resources; project administration. **Anna E. Marneth**: Methodology; investigation; data curation; formal analysis; writing—review and editing; conceptualization; validation; project administration; resources. **Megan Bywater**: Conceptualization; methodology; supervision; writing—review and editing; project administration; data curation; investigation. **Steven W. Lane**: Conceptualization; supervision; funding acquisition; project administration; resources; writing—review and editing; methodology.

## CONFLICT OF INTEREST STATEMENT

A.M. has received research funding from Morphic and Incyte and has consulted for Cellarity. M.J.B. has received research funding from BMS and Cylene Pharmacueticals for unrelated projects. S.W.L. has received research funding from BMS for unrelated projects and has consulted for Novartis, AbbVie, and GSK.

## FUNDING

A.M. acknowledges funding from NIH NHLBI (R01HL131835) and the Department of Defense Congressionally Directed Medical Research Programs (W81XWH2110909). A.E.M. acknowledges funding from the US Department of Defense (Horizon Award W81XWH‐20‐1‐0904). S.W.L. acknowledges funding from NHMRC investigator grant (1195987). Open access publishing facilitated by The University of Queensland, as part of the Wiley ‐ The University of Queensland agreement via the Council of Australian University Librarians.

## Supporting information


**Supplemental Figure 1:** ***ZRSR2*** **mutation profiles in patients with myeloproliferative neoplasm (MPN) or myelodysplastic neoplasm (MDS).** Localization of mutations in the *ZRSR2* gene identified in patients with MPN or MDS, along with the positions of three independent sgRNAs designed to target Exon 1 or Exon 8 of *ZRSR2* in human megakaryoblastic cells (SET‐2 and CHRF‐288‐11). Mutation profiles were generated based on public data from the Catalogue of Somatic Mutations in Cancer (COSMIC) using St. Jude Cloud Pecan (https://pecan.stjude.cloud/).

**Supplemental Figure 2:** ***ZRSR2*** **editing in human megakaryoblastic cell lines.** (A) ZRSR2 variant allele frequency (VAF) in SET‐2 cells targeted by three individual guide RNAs targeting Exon 1 (sg1, sg3) or Exon 8 of ZRSR2 (sg2). (B) ZRSR2 protein levels in transduced SET‐2 cells disrupting ZRSR2 using three independent sgRNAs (sg1, sg2, and sg3) or two independent non‐targeting guides (NTG1 and NTG2). SET cells transduced with Cas9 only serve as an additional control. Cells were collected on Day 17 posttransduction (biological replicate 1) or on Day 20 posttransduction (biological Replicate 2). (C) *ZRSR2* mutation types of each selected cell clone of transduced CHRF‐288‐11 cells. (D) ZRSR2 protein levels in single‐cell clones of transduced CHRF‐288‐11 cells, where ZRSR2 was disrupted using three independent sgRNAs (sg1, sg2, and sg3) or two independent non‐targeting guides (NTG1 and NTG2) from two independent experiments (Replicate 1 and Replicate 2). HEK293T cells transduced with pRRL‐ZRSR2 served as a positive control. The selected single‐cell clones of CHRF‐288‐11 ZRSR2 KO cells or NTG controls used for RNA sequencing are highlighted in red.

**Supplemental Figure 3:** ***Zrsr2*** **editing in a mouse model of** ***Jak2*** ^**V617F**^ **‐driven murine MPN.** (A) Engraftment of transplanted Lineage^−^ Sca‐1^+^ Kit^+^ (LSK) cells in recipients was confirmed by assessing the frequency of mCherry‐expressing cells in peripheral blood at Week 4 posttransplant, in mice transplanted with Jak2^V617F^ cells expressing either sgZrsr2 (sgZrsr2) or empty vector (EV) control. (B) Confirmation of CreER activity in engrafted LSK cells based on the presence of cells expressing both mCherry and GFP fluorescent proteins in peripheral blood at Week 4 post‐tamoxifen administration. (C) Detection of a *Jak2* ^V617F^ unique PCR amplicon using unsorted peripheral blood mononuclear cells collected from mice at the moribund stage. (D) *Jak2* ^V617F^ VAF in sorted mCherry^+,^ GFP^+^ LSK cells from the bone marrow of *Jak2* ^V617F^‐sgZrsr2 recipient mice, collected as the mice became moribund. (E) DNA sequence chromatograms of the *Zrsr2* amplified region spanning the cut site, derived from bulk bone marrow cells from recipient mice at the moribund stage. (F) *Zrsr2* VAF in sorted mCherry^+^, GFP^+^ LSK cells from the bone marrow of *Jak2* ^V617F^‐sgZrsr2 recipient mice at the moribund stage. (G) Western blot of 3XFLAG‐tagged WT and truncated ZRSR2 (c.210delA, ZRSR2^R72fs*11^) protein overexpression in HEK293T cells. The c.210delA in *Zrsr2* is present in all mice, accounting for 35%–46% of all *Zrsr2* mutations. Proteins were detected using an anti‐FLAG antibody in total cell lysates. Data are from two independent experiments.

Supplemental Figure 4: *ZRSR2* loss causes aberrant splicing in human megakaryoblastic cell lines. (A) Gene set enrichment analysis (GSEA) plot on gene sets containing U12‐type introns in SET‐2 *ZRSR2* KO cells versus non‐targeting SET‐2 control cells showing decreased expression of U12‐containing genes in SET‐2 *ZRSR2* KO as compared to control cells (left panel). GSEA plot on gene sets containing U12‐type introns in CHRF‐288‐11 *ZRSR2* KO cells versus non‐targeting CHRF‐288‐11 control cells showing increased expression of U12‐containing genes in CHRF‐288‐11 *ZRSR2* KO as compared to control cells (right panel). (B) GSEA of MK‐primed gene sets shows upregulation in CHRF‐288‐11 *ZRSR2* KO as compared to control cells (upper panel). GSEA of hallmark E2F target genes shows upregulation in CHRF‐288‐11 *ZRSR2* KO as compared to control cells (bottom panel).

**Supplemental Figure 5: The disease phenotype of** ***Jak2*** ^**V617F**^ **‐sgZrsr2 mice is not affected by gender.** (A) Peripheral blood parameters in male or female mice transplanted with *Jak2* ^V617F^ cells expressing sgZrsr2: leukocyte number, hematocrit, and platelet number. (B) Bone marrow cellularity and spleen weights in male or female mice transplanted with *Jak2* ^V617F^ cells expressing sgZrsr2. (C) Percentages of erythroid progenitors in the spleen of male or female mice transplanted with *Jak2* ^V617F^ cells expressing sgZrsr2. CD71^high^/Ter119^low^, CD71^high^/Ter119^high^, CD71^dim^/Ter119^high^, and CD71^−^/Ter119^high^ were defined as Region I (RI), Region II (RII), Region III (RIII), and Region IV (RIV), respectively. In erythropoiesis, these regions are enriched in Pro, Baso, Poly, and Ortho erythroblasts, respectively. (D) Kaplan–Meier survival curve of male or female mice transplanted with *Jak2* ^V617F^ cells expressing sgZrsr2. Green dotted lines show physiological data in C57BL/6 J mice (www.jax.org/phenome). Data were collected as the mice became moribund and were pooled from three independent experiments (*n* = 2–5 mice/genotype/experiment). Results are presented as mean ± s.e.m. Each circle represents one mouse, and P‐values are indicated.

**Supplemental Figure 6:** ***Zrsr2*** **loss does not accelerate** ***Jak2*** ^**V617F**^ **‐driven MPN.** (A) Proportions of major mature hematopoietic cell lineages: B cells (B220^+^), T cells (CD3^+^), and myeloid cells (Gr1^+^) in transduced peripheral blood (PB) cells (mCherry^+^, GFP^+^) from Jak2^V617F^‐sgZrsr2 and Jak2^V617F^‐EV recipient mice. (B) Frequency of GFP and mCherry double‐positive cells (mCherry^+^, GFP^+^) in major mature hematopoietic cell lineages: B cells (B220^+^), T cells (CD3^+^), and myeloid cells (Gr1^+^) in *Jak2* ^V617F^‐sgZrsr2 and *Jak2* ^V617F^‐EV recipient mice. (C) Proportions of progenitor cells (c‐Kit^+^) in transduced PB cells (mCherry^+^, GFP^+^) from Jak2^V617F^‐sgZrsr2 and Jak2^V617F^‐EV recipient mice. (D) Frequency of GFP and mCherry double‐positive cells (mCherry^+^, GFP^+^) in progenitor cells (c‐Kit^+^) in *Jak2* ^V617F^‐sgZrsr2 and *Jak2* ^V617F^‐EV recipient mice. (E) Frequency of GFP and mCherry double‐positive cells (mCherry^+^, GFP^+^) in the HSPC compartment: megakaryocytic progenitor (MkP), common myeloid progenitor (CMP), granulocyte/macrophage progenitor (GMP), megakaryocytic/erythroid progenitor (MEP), and long‐term HSC (LT‐HSC) in *Jak2* ^V617F^‐sgZrsr2 and *Jak2* ^V617F^‐EV recipient mice. Murine HSPC compartment subsets were defined as CMP (Lineage^−^Sca‐1^−^cKit^+^CD34^+^FcγR^−^), GMP (Lineage^−^Sca‐1^−^cKit^+^CD34^+^FcγR^+^), MEP (Lineage^−^Sca‐1^−^cKit^+^CD34^−^FcγR^−^), MkP (Lineage^−^Sca‐1^−^cKit^+^CD150^+^CD41^+^), and LT‐HSCs (Lineage^−^Sca‐1^+^cKit^+^CD150^+^CD48^−^). Lineage markers include CD3e, B220, Ter‐119, Mac‐1, Gr‐1, and CD5. Data are pooled from three independent experiments (*n* = 2–5 mice/genotype/experiment) and represent the mean ± s.e.m. Each circle represents one mouse, and P‐values are indicated. (F) Hematoxylin and eosin‐stained histopathological sections of the spleen (4× or 20× magnification) and the bone marrow (10× or 40× magnification) from representative *Jak2* ^V617F^‐sgZrsr2 and *Jak2* ^V617F^‐EV recipient mice.

**Supplemental Figure 7:** ***Zrsr2*** **loss causes aberrant splicing in** ***Jak2*** ^**V617F**^ **‐driven murine MPN.** (A) Lists of overlapping genes with IRs detected in LSK cells of *Jak2* ^V617F^‐sgZrsr2 recipient mice (total 130 differential RIs) or in *ZRSR2* KO human megakaryoblastic cell lines: ZRSR2 KO SET‐2 cells (total 54 differential RIs) and ZRSR2 KO CHRF‐288‐11 cells (total 132 differential RIs) (FDR < 0.05). (B) GSEA of MK‐primed and LSK gene sets shows upregulation in transduced LSK cells from *Jak2* ^V617F^‐sgZrsr2 recipient mice compared to *Jak2* ^V617F^‐EV recipient mice. NES normalized enrichment score (NES). NES normalized enrichment score (NES). (C) *Zrsr1* gene expression in sorted mCherry^+^, GFP^+^ LSK cells from the bone marrow of *Jak2* ^V617F^‐sgZrsr2 recipient mice or EV control recipient mice, collected as the mice became moribund.

Supplemental Figure 8: Correlation matrix of ZRSR2 VAF and blood parameters in DFCI cohort. Correlation matrix of ZRSR2 VAF with blood parameters—hemoglobin (HGB), white blood cell (WBC) counts, and platelet counts—using DFCI cohort (upper panel). The correlation was assessed using Pearson correction coefficient (*r*) with a two‐tailed test, and results are listed in the table (lower panel).

Supporting Information.

Supporting Information.

Supporting Information.

Supporting Information.

Supporting Information.

## Data Availability

Data are available in NCBI's Gene Expression Omnibus (GEO) for RNA‐seq data of human megakaryoblastic cell lines and murine hematopoietic stem cells under accession number GSE225396 (https://www.ncbi.nlm.nih.gov/geo/query/acc.cgi).
