# Supplementary figures and images for: ZRSR2 loss causes aberrant splicing in JAK2V617F‐driven myeloproliferative neoplasm but is not sufficient to drive disease progression
Source: Hemasphere. 2025 Sep 16;9(9):e70225. doi: 10.1002/hem3.70225 (PMC12439484; doi:10.1002/hem3.70225)

Figure S1

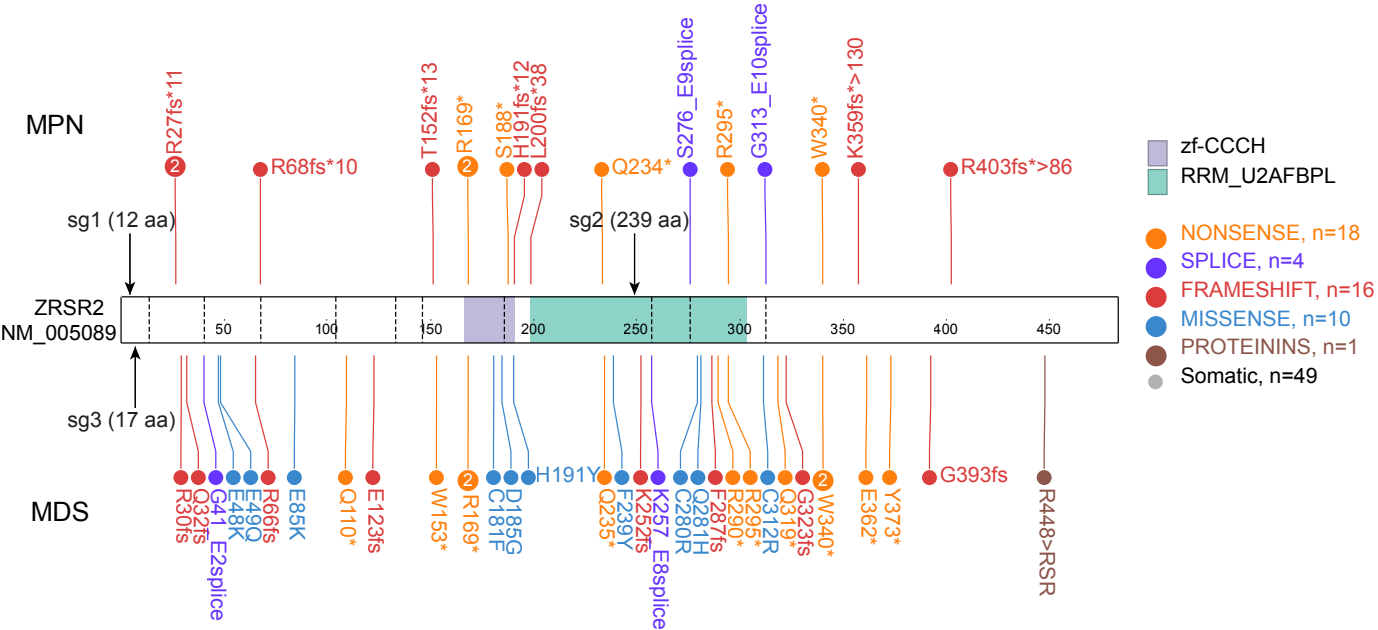

Supplement: Supplementary file 1 — Supplemental Figure 1: ZRSR2 mutation profiles in patients with myeloproliferative neoplasm (MPN) or myelodysplastic neoplasm (MDS). Localization of mutations in the ZRSR2 gene identified in patients with MPN or MDS, along with the positions of three independent sgRNAs designed to target Exon 1 or Exon 8 of ZRSR2 in human megakaryoblastic cells (SET‐2 and CHRF‐288‐11). Mutation profiles were generated based on public data from the Catalogue of Somatic Mutations in Cancer (COSMIC) using St. Jude Cloud Pecan (https://pecan.stjude.cloud/). [file HEM3-9-e70225-s009.pdf]

Figure S2

ZRSR2 editing in SET-2 cell pool

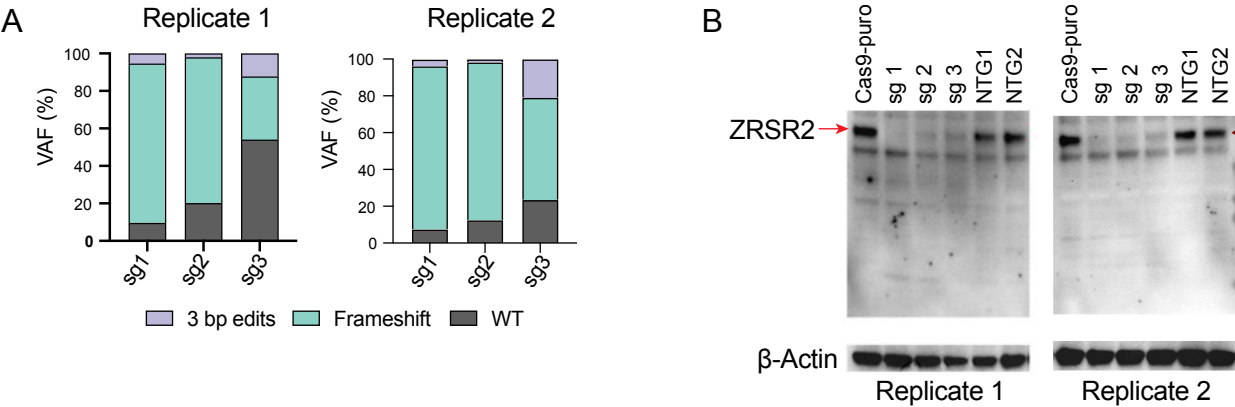

ZRSR2 editing in single cell clones of CHRF-288-11

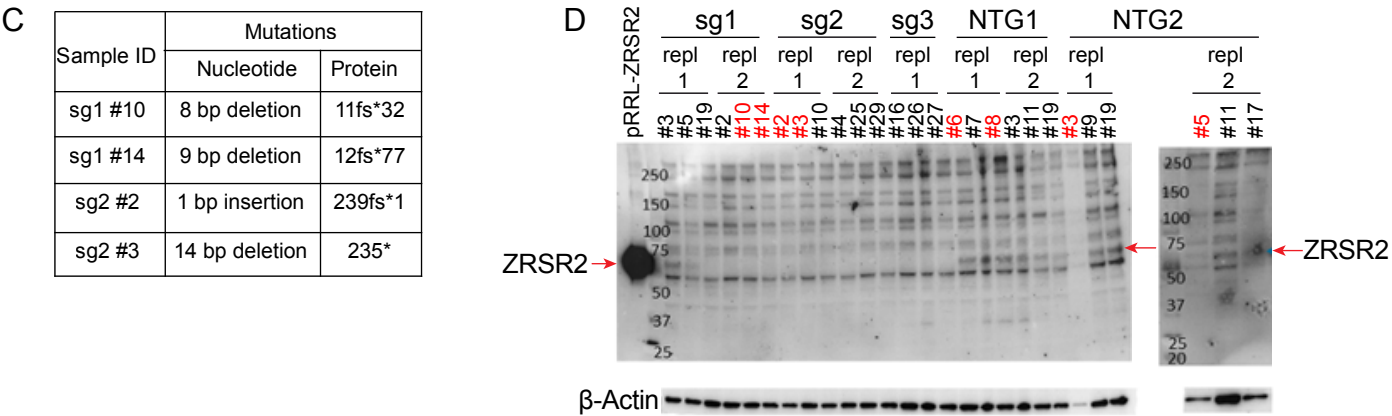

Supplement: Supplementary file 2 — Supplemental Figure 2: ZRSR2 editing in human megakaryoblastic cell lines. (A) ZRSR2 variant allele frequency (VAF) in SET‐2 cells targeted by three individual guide RNAs targeting Exon 1 (sg1, sg3) or Exon 8 of ZRSR2 (sg2). (B) ZRSR2 protein levels in transduced SET‐2 cells disrupting ZRSR2 using three independent sgRNAs (sg1, sg2, and sg3) or two independent non‐targeting guides (NTG1 and NTG2). SET cells transduced with Cas9 only serve as an additional control. Cells were collected on Day 17 posttransduction (biological replicate 1) or on Day 20 posttransduction (biological Replicate 2). (C) ZRSR2 mutation types of each selected cell clone of transduced CHRF‐288‐11 cells. (D) ZRSR2 protein levels in single‐cell clones of transduced CHRF‐288‐11 cells, where ZRSR2 was disrupted using three independent sgRNAs (sg1, sg2, and sg3) or two independent non‐targeting guides (NTG1 and NTG2) from two independent experiments (Replicate 1 and Replicate 2). HEK293T cells transduced with pRRL‐ZRSR2 served as a positive control. The selected single‐cell clones of CHRF‐288‐11 ZRSR2 KO cells or NTG controls used for RNA sequencing are highlighted in red. [file HEM3-9-e70225-s002.pdf]

Figure S3

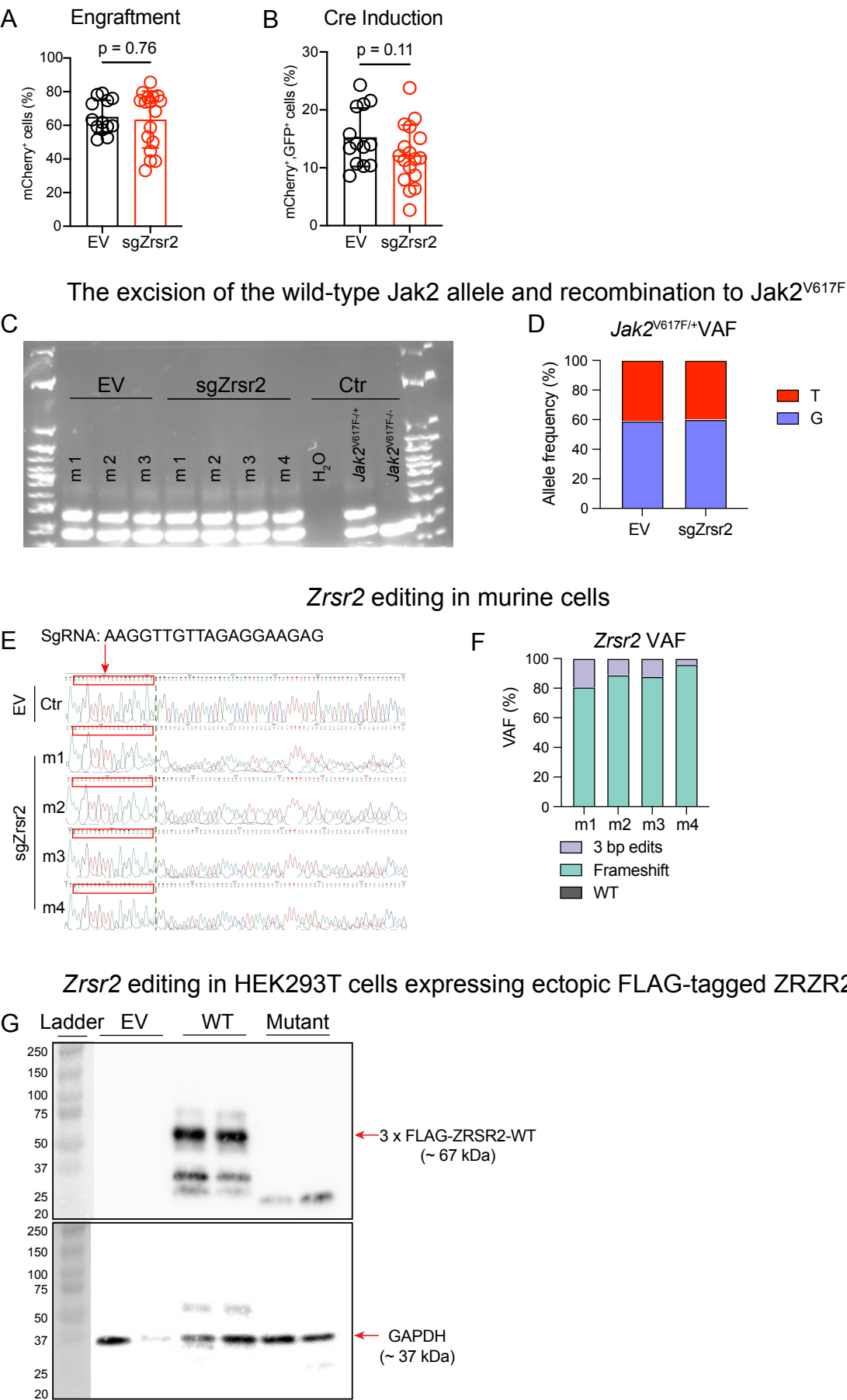

Supplement: Supplementary file 3 — Supplemental Figure 3: Zrsr2 editing in a mouse model of Jak2 V617F ‐driven murine MPN. (A) Engraftment of transplanted Lineage− Sca‐1+ Kit+ (LSK) cells in recipients was confirmed by assessing the frequency of mCherry‐expressing cells in peripheral blood at Week 4 posttransplant, in mice transplanted with Jak2V617F cells expressing either sgZrsr2 (sgZrsr2) or empty vector (EV) control. (B) Confirmation of CreER activity in engrafted LSK cells based on the presence of cells expressing both mCherry and GFP fluorescent proteins in peripheral blood at Week 4 post‐tamoxifen administration. (C) Detection of a Jak2 V617F unique PCR amplicon using unsorted peripheral blood mononuclear cells collected from mice at the moribund stage. (D) Jak2 V617F VAF in sorted mCherry+, GFP+ LSK cells from the bone marrow of Jak2 V617F‐sgZrsr2 recipient mice, collected as the mice became moribund. (E) DNA sequence chromatograms of the Zrsr2 amplified region spanning the cut site, derived from bulk bone marrow cells from recipient mice at the moribund stage. (F) Zrsr2 VAF in sorted mCherry+, GFP+ LSK cells from the bone marrow of Jak2 V617F‐sgZrsr2 recipient mice at the moribund stage. (G) Western blot of 3XFLAG‐tagged WT and truncated ZRSR2 (c.210delA, ZRSR2R72fs*11) protein overexpression in HEK293T cells. The c.210delA in Zrsr2 is present in all mice, accounting for 35%–46% of all Zrsr2 mutations. Proteins were detected using an anti‐FLAG antibody in total cell lysates. Data are from two independent experiments. [file HEM3-9-e70225-s013.pdf]

Figure S4

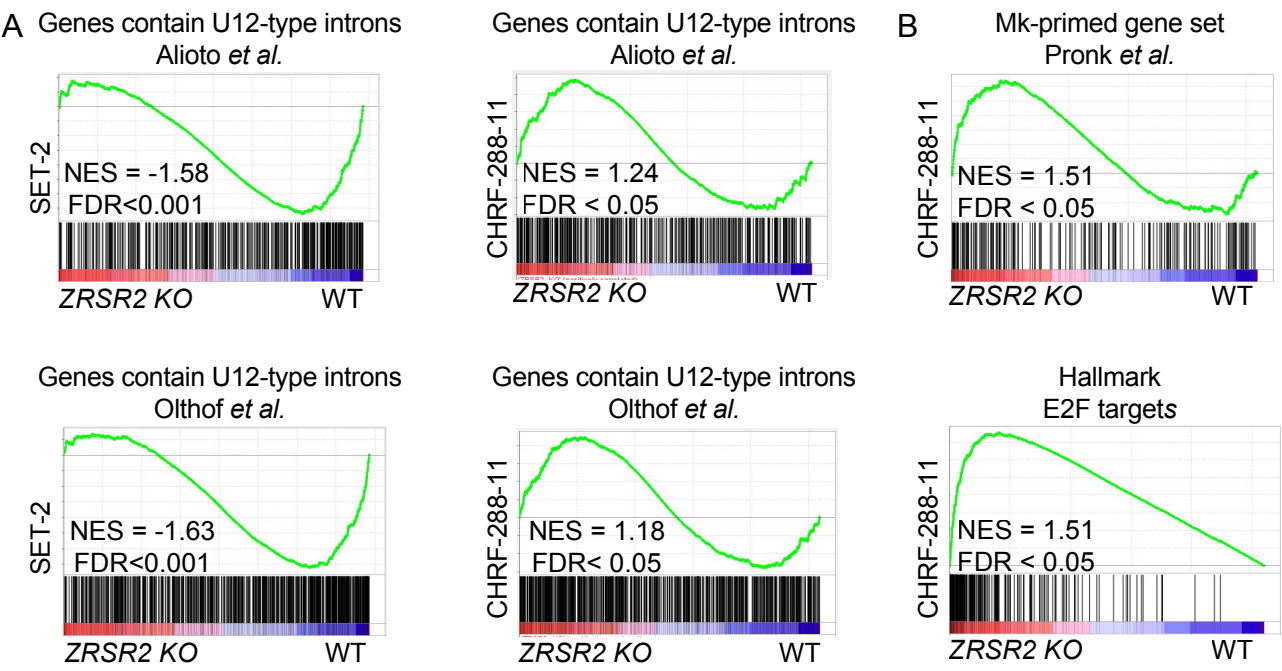

Supplement: Supplementary file 4 — Supplemental Figure 4: ZRSR2 loss causes aberrant splicing in human megakaryoblastic cell lines. (A) Gene set enrichment analysis (GSEA) plot on gene sets containing U12‐type introns in SET‐2 ZRSR2 KO cells versus non‐targeting SET‐2 control cells showing decreased expression of U12‐containing genes in SET‐2 ZRSR2 KO as compared to control cells (left panel). GSEA plot on gene sets containing U12‐type introns in CHRF‐288‐11 ZRSR2 KO cells versus non‐targeting CHRF‐288‐11 control cells showing increased expression of U12‐containing genes in CHRF‐288‐11 ZRSR2 KO as compared to control cells (right panel). (B) GSEA of MK‐primed gene sets shows upregulation in CHRF‐288‐11 ZRSR2 KO as compared to control cells (upper panel). GSEA of hallmark E2F target genes shows upregulation in CHRF‐288‐11 ZRSR2 KO as compared to control cells (bottom panel). [file HEM3-9-e70225-s003.pdf]

Figure S5

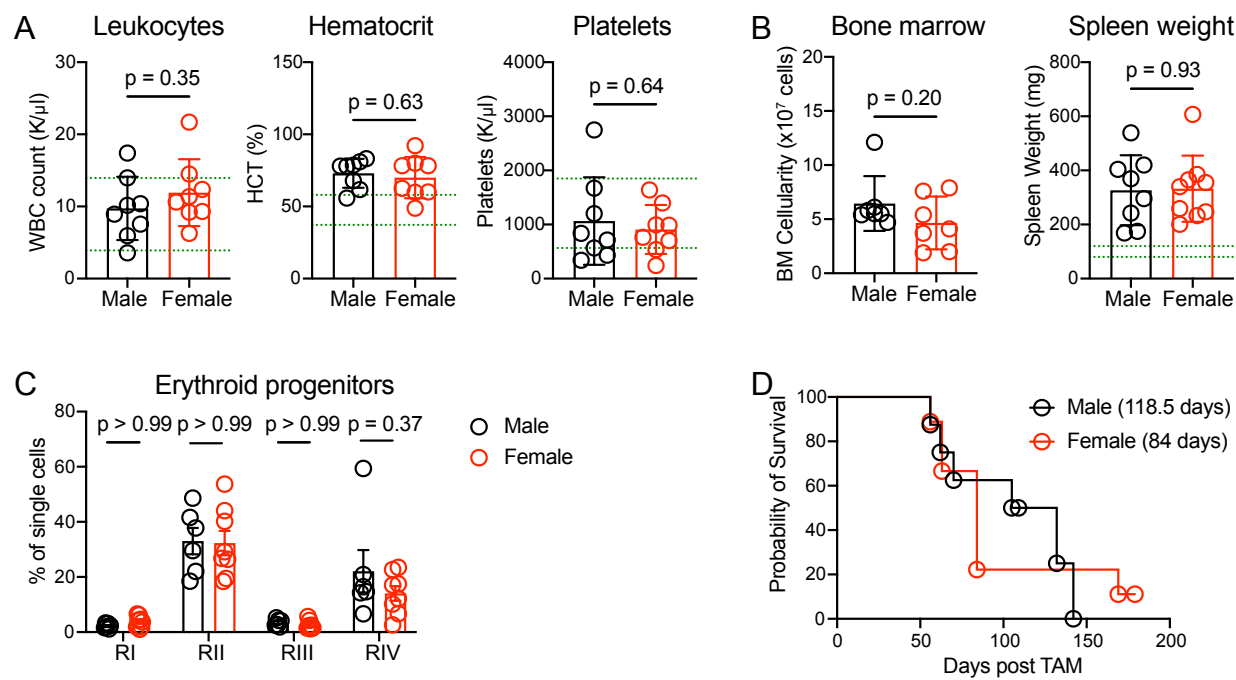

Supplement: Supplementary file 5 — Supplemental Figure 5: The disease phenotype of Jak2 V617F ‐sgZrsr2 mice is not affected by gender. (A) Peripheral blood parameters in male or female mice transplanted with Jak2 V617F cells expressing sgZrsr2: leukocyte number, hematocrit, and platelet number. (B) Bone marrow cellularity and spleen weights in male or female mice transplanted with Jak2 V617F cells expressing sgZrsr2. (C) Percentages of erythroid progenitors in the spleen of male or female mice transplanted with Jak2 V617F cells expressing sgZrsr2. CD71high/Ter119low, CD71high/Ter119high, CD71dim/Ter119high, and CD71−/Ter119high were defined as Region I (RI), Region II (RII), Region III (RIII), and Region IV (RIV), respectively. In erythropoiesis, these regions are enriched in Pro, Baso, Poly, and Ortho erythroblasts, respectively. (D) Kaplan–Meier survival curve of male or female mice transplanted with Jak2 V617F cells expressing sgZrsr2. Green dotted lines show physiological data in C57BL/6 J mice (www.jax.org/phenome). Data were collected as the mice became moribund and were pooled from three independent experiments (n = 2–5 mice/genotype/experiment). Results are presented as mean ± s.e.m. Each circle represents one mouse, and P‐values are indicated. [file HEM3-9-e70225-s004.pdf]

Figure S6

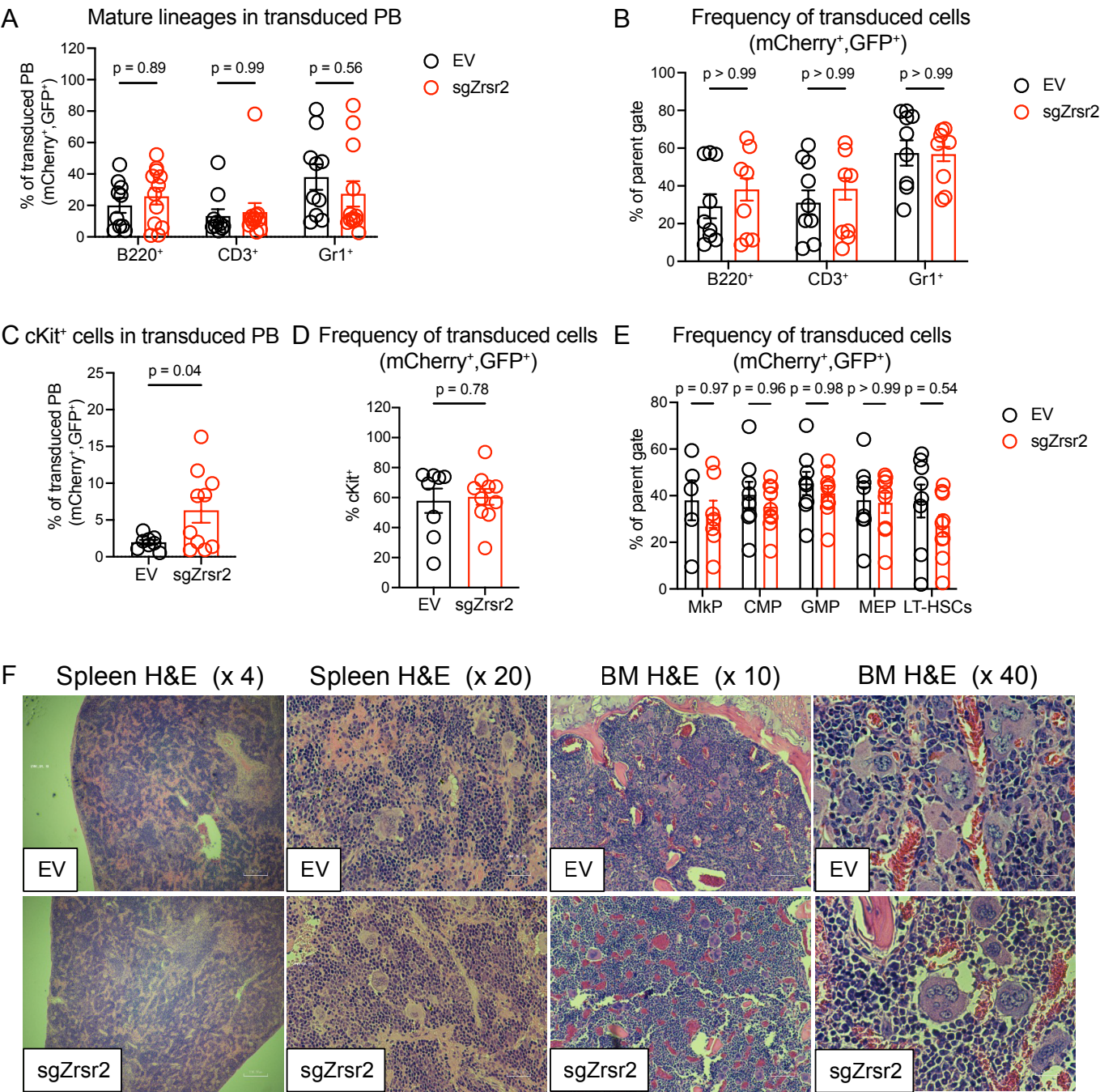

Supplement: Supplementary file 6 — Supplemental Figure 6: Zrsr2 loss does not accelerate Jak2 V617F ‐driven MPN. (A) Proportions of major mature hematopoietic cell lineages: B cells (B220+), T cells (CD3+), and myeloid cells (Gr1+) in transduced peripheral blood (PB) cells (mCherry+, GFP+) from Jak2V617F‐sgZrsr2 and Jak2V617F‐EV recipient mice. (B) Frequency of GFP and mCherry double‐positive cells (mCherry+, GFP+) in major mature hematopoietic cell lineages: B cells (B220+), T cells (CD3+), and myeloid cells (Gr1+) in Jak2 V617F‐sgZrsr2 and Jak2 V617F‐EV recipient mice. (C) Proportions of progenitor cells (c‐Kit+) in transduced PB cells (mCherry+, GFP+) from Jak2V617F‐sgZrsr2 and Jak2V617F‐EV recipient mice. (D) Frequency of GFP and mCherry double‐positive cells (mCherry+, GFP+) in progenitor cells (c‐Kit+) in Jak2 V617F‐sgZrsr2 and Jak2 V617F‐EV recipient mice. (E) Frequency of GFP and mCherry double‐positive cells (mCherry+, GFP+) in the HSPC compartment: megakaryocytic progenitor (MkP), common myeloid progenitor (CMP), granulocyte/macrophage progenitor (GMP), megakaryocytic/erythroid progenitor (MEP), and long‐term HSC (LT‐HSC) in Jak2 V617F‐sgZrsr2 and Jak2 V617F‐EV recipient mice. Murine HSPC compartment subsets were defined as CMP (Lineage−Sca‐1−cKit+CD34+FcγR−), GMP (Lineage−Sca‐1−cKit+CD34+FcγR+), MEP (Lineage−Sca‐1−cKit+CD34−FcγR−), MkP (Lineage−Sca‐1−cKit+CD150+CD41+), and LT‐HSCs (Lineage−Sca‐1+cKit+CD150+CD48−). Lineage markers include CD3e, B220, Ter‐119, Mac‐1, Gr‐1, and CD5. Data are pooled from three independent experiments (n = 2–5 mice/genotype/experiment) and represent the mean ± s.e.m. Each circle represents one mouse, and P‐values are indicated. (F) Hematoxylin and eosin‐stained histopathological sections of the spleen (4× or 20× magnification) and the bone marrow (10× or 40× magnification) from representative Jak2 V617F‐sgZrsr2 and Jak2 V617F‐EV recipient mice. [file HEM3-9-e70225-s011.pdf]

Figure S7

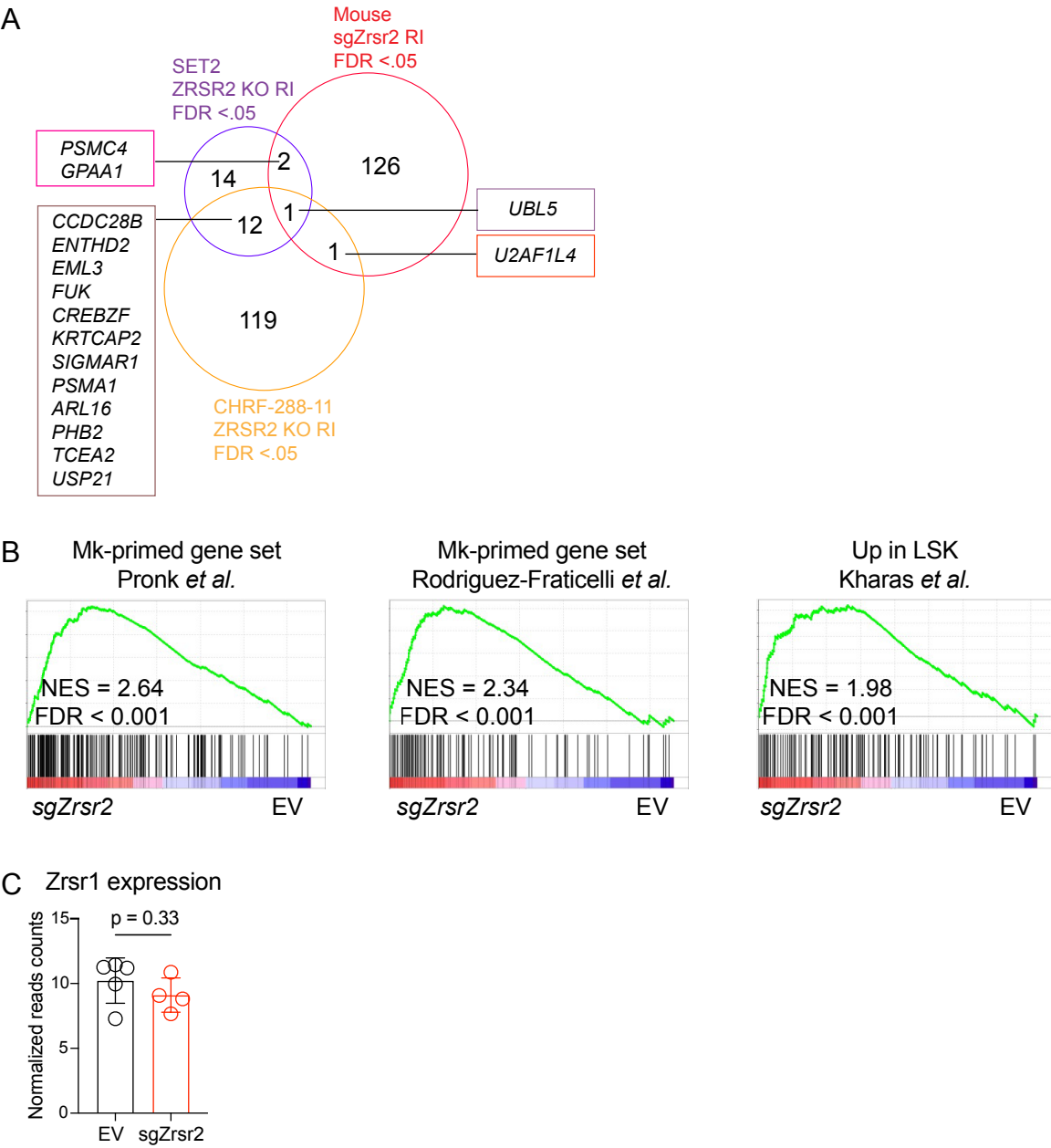

Supplement: Supplementary file 7 — Supplemental Figure 7: Zrsr2 loss causes aberrant splicing in Jak2 V617F ‐driven murine MPN. (A) Lists of overlapping genes with IRs detected in LSK cells of Jak2 V617F‐sgZrsr2 recipient mice (total 130 differential RIs) or in ZRSR2 KO human megakaryoblastic cell lines: ZRSR2 KO SET‐2 cells (total 54 differential RIs) and ZRSR2 KO CHRF‐288‐11 cells (total 132 differential RIs) (FDR < 0.05). (B) GSEA of MK‐primed and LSK gene sets shows upregulation in transduced LSK cells from Jak2 V617F‐sgZrsr2 recipient mice compared to Jak2 V617F‐EV recipient mice. NES normalized enrichment score (NES). NES normalized enrichment score (NES). (C) Zrsr1 gene expression in sorted mCherry+, GFP+ LSK cells from the bone marrow of Jak2 V617F‐sgZrsr2 recipient mice or EV control recipient mice, collected as the mice became moribund. [file HEM3-9-e70225-s010.pdf]

Figure S8

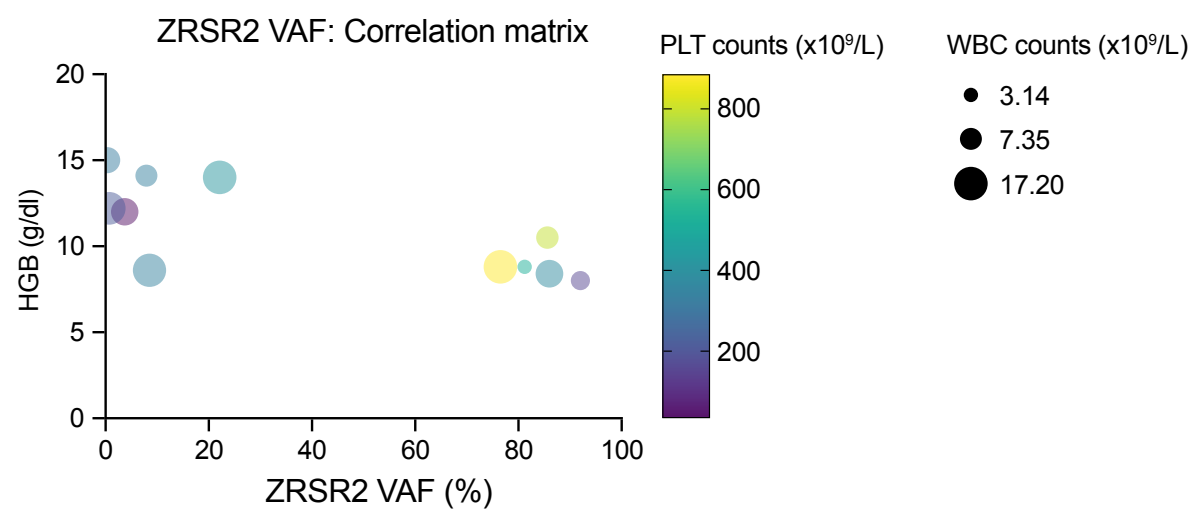

|           |           | ZRSR2 VAF | HGB   | WBC counts | PLT counts |
|-----------|-----------|-----------|-------|------------|------------|
| ZRSR2 VAF | Pearson r | 1         | -0.74 | -0.43      | 0.53       |
|           | P values  |           | 0.01  | 0.18       | 0.10       |

Supplement: Supplementary file 8 — Supplemental Figure 8: Correlation matrix of ZRSR2 VAF and blood parameters in DFCI cohort. Correlation matrix of ZRSR2 VAF with blood parameters—hemoglobin (HGB), white blood cell (WBC) counts, and platelet counts—using DFCI cohort (upper panel). The correlation was assessed using Pearson correction coefficient (r) with a two‐tailed test, and results are listed in the table (lower panel). [file HEM3-9-e70225-s007.pdf]
