# Supplementary material for: ZRSR2 loss causes aberrant splicing in JAK2V617F‐driven myeloproliferative neoplasm but is not sufficient to drive disease progression
Source: Hemasphere. 2025 Sep 16;9(9):e70225. doi: 10.1002/hem3.70225 (PMC12439484; doi:10.1002/hem3.70225)
Supplement: Supplementary file 9 — Supporting Information. [file HEM3-9-e70225-s012.docx]

**Materials and Methods**

***ZRSR2* knockout (KO) human megakaryoblastic cell lines**

PLKO5 GFP plasmids containing sgRNAs targeting human *ZRSR2* (sg1: TGACGTTTCCCGAGAAACCA, sg2: ACAGTTCCTAGACTTCTATG, sg3: CTTCCAGCCACAAAAAGTAC) and non-targeting sgRNAs control (NTG1: GACGGAGGCTAAGCGTCGCAA, NTG2: GCGCTTCCGCGGCCCGTTCAA) were a kind gift from Dr. Togami.^1^ 293T cells were cultured in Dulbecco's Modified Eagle Medium (DMEM) with high glucose (Fisher Scientific), 10% fetal bovine serum (Omega Scientific), and 1% penicillin-Streptomycin solution (Caisson Labs). 293T cells were transfected with Cas9-puro plasmid or pLKO5 GFP, together with pCMV-VSVg and psPAX2 using polyethylenimine (PEI, Sigma-Aldrich). Medium was refreshed 16 hours post transfection and viral supernatant harvested 24 to 48 hours later. Viral particles were then spun down onto tissue culture plates coated with retronectin, virus-depleted media removed, and followed by a spin down of SET-2 or CHRF-288-11 cells onto virally coated retronectin. The cells were incubated overnight, followed by a second round of transduction. Cells expressing Cas9 were selected using 2 µg/ml puromycin, followed by transduction with sgRNA-targeting virus. Individual transduced CHRF-288-11 cells were sorted and clonally expanded for further culture.

**Mice**

Alleles used to generate the compound genotype *Jak2^fl-V617F /+^; Rosa26^lsl-Cas9-T2A-GFP/creER^* on a C57BL/6 background are previously described.^2,3^ Wild-type C57BL/6 mice were purchased from Animal Resource Center (ARC), Australia. Mice were maintained in pathogen-free facilities at QIMR Berghofer Medical Research Institute, ethics protocol A2109-620.

**Generation of *Zrsr2* loss in *Jak2*^V617F^-driven MPN mice**

The lentiviral vector-pLKO5.sgRNA.EFS.mCherry was previously described.^4^ Single guide RNA targeting exon 3 of mouse *Zrsr2* (sgRNA: AAGGTTGTTAGAGGAAGAG) was cloned into pLKO5.sgRNA.EFS.mCherry and validated by Sanger sequencing.^5^ The lentiviral backbone vector containing sgRNA targeting mouse *Zrsr2* (sgZrsr2) or empty vector control without insertion of sgRNA region (EV), together with packaging plasmids pMD2.G (Addgene plasmid #12259) and psPAX2 (Addgene plasmid #12260) were transfected into HEK293T cells (ATCC CRL-3216) using Fugene 6 (Promega). Medium was refreshed 16 hours post transfection. Lentiviral particles were harvested 24 hours and 48 hours after transfection and concentrated via ultracentrifugation.

Sorted Lineage^-^Sca-1^+^c-Kit^+^ (LSK) cells were recovered for two hours at 37°C in StemSpan™ Serum-Free Expansion Medium II (SFEM II) (Stem Cell) containing 10 ng/mL recombinant murine TPO (Lonza Bioscience), 50 ng/mL recombinant murine SCF (Lonza Bioscience), and 10 ng/mL recombinant murine G-SCF (Lonza Bioscience) prior to transduction with a lentivirus harboring sgZrsr2 or EV overnight, with a target multiplicity of infection (MOI) of 20. Cells were then cultured in fresh media, as detailed above, for 24h before transplantation. Recipient mice, C57BL/6, received a lethal dose of irradiation, administered over 2 doses of 550 cGy, at least three hours apart within 24 hours of transplantation. Cells (50,000-200,000 infected LSKs/mouse mixed with 30,000 helper C57BL/6 donor BM/mouse) were administered by lateral tail vein injection. Transplanted mice were maintained on antibiotic (Baytril) water for two weeks. Four weeks post-transplant, peripheral blood (PB) was sampled by submandibular bleeding to confirm successful engraftment with the presence of mCherry expressing cells via flow cytometry. Mice were maintained on Tamoxifen Chow (400mg/kg, Specialty Feeds) for two weeks to induce CreER activity and expression of the conditional alleles, *Jak2*^V617F^, GFP and Cas9, enabling the editing of *Zrsr2,* before returning to normal chow. Four weeks after commencement of Tamoxifen administration, PB was sampled to confirm the successful induction of CreER and Cas9 by confirming the presence of GFP and mCherry positive cells via flow cytometry.

**Blood count analysis**

Peripheral blood (PB) was collected by submandibular vein bleeding into EDTA-coated tubes, and analyzed on a Hemavet analyzer (Model 950, Drew scientific).

**Histology**

Hematoxylin and eosin (H&E) staining were performed on formalin-fixed and paraffin-embedded tissues in histology facility at QIMR Berghofer Medical Research Institute.

**Fluorescence-activated Cell Sorting (FACS) and Flow Cytometry**

BM cells from *Jak2^fl-V617F /+^; Rosa26^lsl-Cas9-T2A-GFP/creER^* male donor mice were stained with a biotin conjugated mature lineage antibody cocktail (CD3e, B220, Ter-119, Mac-1, Gr-1, and CD5). Lineage-positive cells were depleted with anti-biotin dynabeads (BD) on magnet as per manufacturer’s instructions. Lineage depleted BM cells were collected and stained with a Streptavidin conjugate and antibodies against mouse Sca-1, and c-Kit. Lineage^-^Sca-1^+^c-Kit^+^ (LSK) cells were sorted using a FACS ARIA IIIu (BD Biosciences).

Surface marker staining was performed on single cell suspensions in PBS with 2% fetal calf serum for 30 minutes at 4 °C using antibodies and dilutions listed in Supplemental Table 1. Flow cytometry was performed on a FACS LSRII Fortessa (BD Biosciences), and results were analyzed using FlowJo software V10.8.1V (Treestar).

**PCR and Sequencing**

Genomic DNA was isolated from human megakaryoblastic cells (QIAamp DNA Blood kits, Qiagen) or murine bone marrow cells (QuickExtract DNA Extraction solution, Illumina) and amplified using primers listed in the Supplemental Table 1.

PCR products of megakaryoblastic cells were subjected to CRISPR next-generation sequencing at the Mass General Hospital Center for computational and integrative biology DNA Core. Murine PCR products were purified with ExoSAP-IT™ PCR Product Cleanup (Thermo Fisher Scientific) followed by Sanger sequencing.

**Overexpression of FLAG-Zrsr2 construct in HEK293T**

As the effect of Cas9 editing on Zrsr2 expression in the BM was unable to be determined by immunoblotting due to the lack of a murine Zrsr2 antibody, we alternatively determined the effect of one of the most frequent mutations c.210delA (35%-46%) in *Zrsr2* that resulted from Cas9-editing on ectopic FLAG-tagged Zrsr2 expression in HEK293T cells. Wild-type or mutant Zrsr2 (c.210delA) cDNA sequences with 3xFLAG epitope were cloned into the expression vector pcDNA3.1 by VectorBuilder. The sequences of the wild-type and mutant Zrsr2 (c.210delA) constructs are provided in the Supplementary text 1. 15 x 10^4^ HEK293T cells were seeded in 2 ml of DMEM with high glucose (Fisher Scientific), 10% fetal bovine serum (Omega Scientific), and 1% penicillin-Streptomycin solution (Caisson Labs) in 6-well plates and allowed to grow at 37°C and 5% CO_2_ overnight. Next day, cell medium was renewed, and cells were transfected with 1μg of construct using FuGENE reagent (Promega) according to the manufacturer's instructions. After 72 h of growth at 37°C and 5% CO_2_, cells were lysed for Western blotting.

**Western blot**

SET-2 cells or CHRF-288-11 cells were lysed in NP40 lysis buffer with complete EDTA-free protease inhibitor cocktail (Sigma-Aldrich) for 20 minutes on a rotator. Lysates were spun down for 20 minutes at full speed and 4^o^C. Supernatant was subjected to Bradford (Bio-Rad) assay to determine protein concentration. Equal amounts of protein (~80µg) were loaded onto a 4-15% TGX Criterion gel (Bio-Rad) and blotted onto PVDF membranes (Cytiva). Membranes were blocked in 5% non-fat dry milk (Santa Cruz Biotechnology) in tris buffered saline with tween (TBST, Santa Cruz Biotechnology) for one hour at room temperature followed by overnight incubation in TBST, 5% milk, and 1:1000 mouse-anti-ZRSR2 (Abnova H00008233-B01P) at 4^o^C. Membranes were washed with TBST, followed by probing with anti-mouse IgG-horseradish peroxidase (1:1000) in TBST for one hour at room temperature. Membranes were washed again and developed using SuperSignal West Dura Chemiluminescent Substrate (Fisher Scientific). Membranes were then incubated for one hour with 1:5000 anti-beta Actin-horseradish peroxidase (Abcam ab20272) in 5% milk TBST at room temperature, washed, and developed as described above.

FLAG-Zrsr2 overexpressed HEK293T cells were lysed using SDS lysis buffer (SDS 1%, Tris 50 mM, glycerol 10%, protease and phosphatase inhibitors (Roche)) at 4°C for 10 minutes. Subsequently, the lysate was subjected to heating at 95°C for 10 minutes, following by maintaining at 4°C until sonication for 15 minutes (D150H, Thermoline Scientific). The protein concentration was determined using a bicinchoninic acid (BCA) protein assay (ThermoFisher; Cat# 23225). A total of 50 μg of protein was loaded onto pre-cast 4-20% polyacrylamide gels (Bio-Rad) and subsequently transferred onto polyvinylidene difluoride (PVDF) membranes (Cytiva). Following a blocking step in 5% skim milk powder in tris-buffered saline (TBS) (20 mM Tris, 0.15 mM NaCl, pH 7.5) or PBS with 0.1% Tween for 1 hour at room temperature, the membrane was incubated with a 1:1000 dilution of primary antibodies: rabbit-anti-FLAG (cell signaling, Cat#14793) at 4°C overnight. Membranes were washed with TBST or PBST, followed by probing with 1:5000 dilution of secondary antibody goat anti-rabbit immunoglobulins/HRP (Agilent, Cat# P044801-2) at room temperature for one hour. Membrane development was carried out using Clarity™ Western ECL Substrate (BioRad, Cat# 1705061) under the iBright CL1500 Chemi Gel Doc (Invitrogen). The product of the housekeeping gene GAPDH was employed as a loading control. After three washes with TBST, the membranes were incubated with a 1:5000 dilution of rabbit-anti-GAPDH at room temperature for one hour, followed by the same procedure as described above.

**RNA sequencing (RNA-Seq) and bioinformatics analysis**

Total RNA was extracted from SET-2 cell pools on day 20 (biological replicate 1) and 25 (biological replicate 2), or from CHRF-288-11 single cell clones post sgRNA transduction, using the RNeasy Mini Kit (Qiagen) with on-column DNAse I digestion as per manufacturer’s protocol. RNA-Seq libraries were prepared using Roche Kapa mRNA HyperPrep sample preparation kits from 200ng of purified total RNA according to the manufacturer’s protocol. The finished dsDNA libraries were quantified by Qubit fluorometer, Agilent TapeStation 2200, and RT-qPCR using the Kapa Biosystems library quantification kit according to manufacturer’s protocols. Uniquely indexed libraries were pooled in equimolar ratios and sequenced on an Illumina NextSeq500 run with paired-end 75bp reads at the Dana-Farber Cancer Institute Molecular Biology Core Facilities.

GFP and mCherry double positive LSK cells from mouse BM were enriched and sorted by BD FACS ARIA IIIu using the same method described above. Total RNA was extracted from sorted LSK cells using Arcturus PicoPure RNA Isolation Kit (Applied Biosystems). Following RNA isolation and quantification, 5 ng of total RNA was used to prepare the cDNA libraries as per the manufacturer’s protocol (NEB Next Ultra II RNA Library Prep Kit for Illumina, New England BioLabs). Briefly, polyA RNA was isolated, fragmented and primed prior to cDNA synthesis, end repair/dA tailing and Illumina adaptor ligation. The resulting products were PCR enriched /indexed with size selection and quantitation before sequencing on the Illumina platform (NextSeq 550) using 150 bp paired end reads.

Sequenced reads were mapped to reference genome and transcript. All RNA-Seq data downstream analysis was performed with R version 3.6.2. RNA-Seq data from human megakaryoblastic cells were generated from SET-2 cell pools (two biological replicates at day 20 and 25) and CHRF-288-11 cells (eight selected single cell clones), both targeted by two individual sgRNAs of *ZRSR2* (sg1 and sg2) or non-targeting sgRNAs (NTG1 and NTG2). Since RNA-Seq showed a batch effect between these experiments, principal component analysis (PCA), hierarchical clustering and gene set enrichment analysis (GSEA v4.2.2) ^6^ were performed on tmm and cpm normalized using edgeR v3.28.1^7^ and batch corrected using the sva v3.34.0 R package with the Combat function.^8^ Differential expression analysis between *ZRSR2* knockout (KO) and control samples using edgeR was performed taking experimental batch as a covariate. RNA-Seq data analysis in mice LSK cells was performed like in human megakaryoblastic cells without batch correction. Intron retention analysis was performed with rMATS v4.0.2,^9^ detailed data listed in Supplemental Table 2. Donor or acceptor splice site motifs of retained introns (False Discovery Rate, FDR<.05) in *ZRSR2* disrupted samples or controls were generated with R package TFBSTools v1.24. Genes containing an U12 intron in mouse or human, respectively were extracted from [U12DB](http://genome.imim.es/cgi-bin/u12db/u12db.cgi)^10^ and [MIDB](https://midb.pnb.uconn.edu/)^11^ on 16/02/2022. The gene sets for GSEA were listed in the Supplemental Table 3.

**Patient Data**

BM or PB samples from patients seen at the Dana-Farber Cancer Institute (DFCI) were screened for MPN-associated mutations in *ASXL1*, *ATM*, *BCOR*, *BRAF*, *BRCC3*, *CALR*, *CBL*, *CDKN2A*, *CEBPA*, *CREBBP*, *CSF3R*, *CTCF*, *CUX1*, *CXCR4*, *DDX41*, *DNMT3A*, *EP300*, *ETC6*, *EZH2*, *FBXW7*, *FLT3*, *GATA2*, *GNAS*, *GNB1*, *IDH1*, *IDH2*, *IKZF1*, *JAK2*, *JAK3*, *KIT*, *KRAS*, *MPL*, *MYD88*, *NF1*, *NFE2*, *NOTCH1*, *NOTCH2*, *NPM1*, *NRAS*, *PDS5B*, *PHF6*, *PPM1D*, *PRPF8*, *PTEN*, *PTPN11*, *RAD21*, *RIT1*, *RUNX1*, *SETBP1*, *SETD2*, *SF3B1*, *SH2B3*, *SMC1A*, *SRSF2*, *STAG2*, *TET2*, *TP53*, *U2AF1*, *WHSC1*, and *ZRSR2* using a clinical in-house next generation sequencing assay.^12^ Patient data was retrieved from the DFCI Hematological Malignancies Data Repository (HMDR) under a DFCI internal review board (IRB) approved protocol (number 21-262).

**Statistical analysis**

Statistical differences were determined by an unpaired t-test for the comparison of two groups. For comparison involving more than two groups and/or two variables, one- or two-way analysis of variance (ANOVA) with Sidak's multiple comparisons correction was used, using GraphPad v. 10. Comparison of categorical variables was performed with Fisher's exact or Chi-square tests. The correlation was assessed using Pearson correction coefficient (r) with two-tailed test. Survival was assessed using Kaplan-Meier analysis and compared using the log-rank test. *P* values of < 0.05 were considered to represent a statistically significant difference.

**Supplemental References:**

1. Togami K, Chung SS, Madan V, et al. Sex-Biased ZRSR2 Mutations in Myeloid Malignancies Impair Plasmacytoid Dendritic Cell Activation and Apoptosis. *Cancer Discov*. Feb 2022;12(2):522-541. doi:10.1158/2159-8290.CD-20-1513

2. Mullally A, Lane SW, Ball B, et al. Physiological Jak2V617F expression causes a lethal myeloproliferative neoplasm with differential effects on hematopoietic stem and progenitor cells. *Cancer Cell*. Jun 15 2010;17(6):584-96. doi:10.1016/j.ccr.2010.05.015

3. Keane TM, Goodstadt L, Danecek P, et al. Mouse genomic variation and its effect on phenotypes and gene regulation. *Nature*. Sep 14 2011;477(7364):289-94. doi:10.1038/nature10413

4. Heckl D, Kowalczyk MS, Yudovich D, et al. Generation of mouse models of myeloid malignancy with combinatorial genetic lesions using CRISPR-Cas9 genome editing. *Nat Biotechnol*. Sep 2014;32(9):941-6. doi:10.1038/nbt.2951

5. Tzelepis K, Koike-Yusa H, De Braekeleer E, et al. A CRISPR Dropout Screen Identifies Genetic Vulnerabilities and Therapeutic Targets in Acute Myeloid Leukemia. *Cell Rep*. Oct 18 2016;17(4):1193-1205. doi:10.1016/j.celrep.2016.09.079

6. Subramanian A, Tamayo P, Mootha VK, et al. Gene set enrichment analysis: a knowledge-based approach for interpreting genome-wide expression profiles. *Proc Natl Acad Sci U S A*. Oct 25 2005;102(43):15545-50. doi:10.1073/pnas.0506580102

7. Robinson MD, McCarthy DJ, Smyth GK. edgeR: a Bioconductor package for differential expression analysis of digital gene expression data. *Bioinformatics*. Jan 1 2010;26(1):139-40. doi:10.1093/bioinformatics/btp616

8. Johnson WE, Li C, Rabinovic A. Adjusting batch effects in microarray expression data using empirical Bayes methods. *Biostatistics*. Jan 2007;8(1):118-27. doi:10.1093/biostatistics/kxj037

9. Shen S, Park JW, Lu ZX, et al. rMATS: robust and flexible detection of differential alternative splicing from replicate RNA-Seq data. *Proc Natl Acad Sci U S A*. Dec 23 2014;111(51):E5593-601. doi:10.1073/pnas.1419161111

10. Alioto TS. U12DB: a database of orthologous U12-type spliceosomal introns. *Nucleic Acids Res*. Jan 2007;35(Database issue):D110-5. doi:10.1093/nar/gkl796

11. Olthof AM, Hyatt KC, Kanadia RN. Minor intron splicing revisited: identification of new minor intron-containing genes and tissue-dependent retention and alternative splicing of minor introns. *BMC Genomics*. Aug 30 2019;20(1):686. doi:10.1186/s12864-019-6046-x

12. Kluk MJ, Lindsley RC, Aster JC, et al. Validation and Implementation of a Custom Next-Generation Sequencing Clinical Assay for Hematologic Malignancies. *J Mol Diagn*. Jul 2016;18(4):507-15. doi:10.1016/j.jmoldx.2016.02.003
