# Supplementary material for: ZRSR2 loss causes aberrant splicing in JAK2V617F‐driven myeloproliferative neoplasm but is not sufficient to drive disease progression
Source: Hemasphere. 2025 Sep 16;9(9):e70225. doi: 10.1002/hem3.70225 (PMC12439484; doi:10.1002/hem3.70225)
Supplement: Supplementary file 10 — Supporting Information. [file HEM3-9-e70225-s008.docx]

**Supplemental Table S1. Primers for PCR**

| Primer name | Sequence 5'-3' |
| --- | --- |
| hZRSR2-sg1_FWD | CCGGCTTTCCGTTTCAAGTC |
| hZRSR2-sg1_REV | TCTTTTCTTCCTGCCACCACA |
| hZRSR2-sg2_FWD | TGCAGGAGGGATGACTATGA |
| hZRSR2-sg2_REV | AGCTCTCTTGTGCCATTAAGT |
| hZRSR2-sg3_FWD | TGCATTGTAGCCGCTGATCG |
| hZRSR2-sg3_REV | CTGACACTGGGGCTTCAAGT |
| mZrsr2-exon3_FWD | AGAAGTGCGGTTAGAGGAATG |
| mZrsr2-exon3_REV | GCCTGTCAGCTAGGGATTTAG |

**Supplemental Table S2. Antibodies for flow cytometry**

| Antibody | Fluorochrome | Clone | Manufacturer | catalogue No. | Dilution |
| --- | --- | --- | --- | --- | --- |
| Gr1 | APC | RB6-8C5 | BioLegend | 108412 | 1:100 |
| B220 (CD45R) | Percp/Cy5.5 | RA3-B2 | BioLegend | 103236 | 1:100 |
| CD3e | PE/Cy7 | 145-2C11 | BioLegend | 100320 | 1:100 |
| CD71 | BV421 | R17217 | BioLegend | 113813 | 1:100 |
| Ter119 | APC | TER119 | BioLegend | 116212 | 1:100 |
| CD41 | BV421 | MWReg30 | BioLegend | 133912 | 1:100 |
| CD41 | BV605 | MWReg30 | BioLegend | 133921 | 1:50 |
| CD117 (c-kit) | PE | 2B8 | BioLegend | 105808 | 1:100 |
| CD117 (c-kit) | PE/Cy7 | 2B8 | BioLegend | 105814 | 1:100 |
| CD117 (c-kit) | APC | 2B8 | BioLegend | 105812 | 1:100 |
| Streptavidin | APC/Cy7 | n/a | BioLegend | 405208 | 1:100 |
| Streptavidin | V450 | n/a | BD | 560797 | 1:100 |
| Streptavidin | BV711 | n/a | BioLegend | 405241 | 1:100 |
| Sca-1 | PE/Cy7 | D7 | BioLegend | 108114 | 1:100 |
| CD150 (SLAM) | BV605 | TC15-12F12.2 | BioLegend | 115927 | 1:100 |
| CD150 (SLAM) | PE/Cy5 | TC15-12F12.2 | BioLegend | 115912 | 1:100 |
| CD34 | Horizon V450 | RAM34 | eBioscience | 48-0341-82 | 1:50 |
| CD34 | Alexa Fluor 700 | RAM34 | Thermo Fisher Scientific | 56-0341-82 | 1:50 |
| CD16/32(FcBlock) | APC/Cy7 | 93 | BioLegend | 101328 | 1:100 |
| CD105 | PE | MJ7/19 | BioLegend | 120408 | 1:50 |
| CD48 | BV421 | HM48-1 | BioLegend | 103427 | 1:100 |
| Sytox-Blue | Pacific Blue | n/a | Invitrogen | s34857 | 1:5000 |
| CD3e | Biotin | 145-2C11 | BioLegend | 100244 |  |
| B220 | Biotin | RA3-6B2 | BioLegend | 103204 |  |
| Ter-119 | Biotin | TER-119 | BioLegend | 116204 |  |
| Mac-1 | Biotin | M1/70 | BioLegend | 101204 |  |
| Gr-1 | Biotin | RB6-8C5 | BioLegend | 108404 |  |
| CD5 | Biotin | 53-7.3 | BioLegend | 100604 |  |

**Supplemental Table S3. Patient database Dana-Farber Cancer Institute (DFCI)**

| **Patient ID** | **Sex** | **First MPN** | **Age first MPN** | **Second MPN** | **Age second MPN** | **Sample ID** | **Pathogenic mutations** |
| --- | --- | --- | --- | --- | --- | --- | --- |
| 1 | Male | PV | 73.3 |  |  | 1 | JAK2 p.V617F, ZRSR2 p.X134_splice |
| 2 | Male | prefibrotic MF | 79.7 |  |  | 1 | ASXL1 p.R965*, JAK2 p.V617F, SRSF2 p.P95H |
|  |  |  |  |  |  | 2 | ASXL1 p.R965*, JAK2 p.V617F, SRSF2 p.P95H, ZRSR2 p.S420Hfs*? |
| 3 | Male | MF | 60.4 |  |  | 1 | ASXL1 p.W583*, JAK2 p.V617F, ZRSR2 p.R182* |
| 4 | Male | ET | 77.0 | MF | 86.5 | 1 | ASXL1 p.E705*, ASXL1 p.G629fs*, JAK2 p.V617F, SF3B1 p.K700E, TET2 p.Q325*, ZRSR2 p.E55* |
|  |  |  |  |  |  | 2 | ASXL1 p.E705*, ASXL1 p.G629fs*, JAK2 p.V617F, SF3B1 p.K700E |
| 5 | Male | MF | 65.4 |  |  | 1 | ASXL1 p.S770*, JAK2 p.V617F |
|  |  |  |  |  |  | 2 | ASXL1 p.S770*, JAK2 p.V617F, ZRSR2 p.Y18* |
| 6 | Male | PV | 41.0 | MF | 55.1 | 1 | JAK2 p.V617F, ZRSR2 p.L37fs* |
| 7 | Male | ET | 67.3 | MF | 77.9 | 1 | JAK2 p.V617F, ZRSR2 p.N171fs* |
| 8 | Male | PV | 57.9 | MF | 75.7 | 1 | JAK2 p.V617F |
|  |  |  |  |  |  | 2 | JAK2 p.V617F, ZRSR2 p.Y227Ifs*15 |
| 9 | Male | ET | 58.1 | MF | 67.0 | 1 | ASXL1 p.Q976*, JAK2 p.V617F, TET2 p.C1298S, TET2 p.L879fs*, TET2 p.Q701fs*, TET2 p.Y791*, ZRSR2 p.H15fs* |
| 10 | Male | PV | 56.4 | MF | 68.5 | 1 | JAK2 p.543_544delED, TET2 p.G520fs*, TET2 p.Q417*, ZRSR2 p.R126* |
|  |  |  |  |  |  | 2 | ASXL1 p.R693*, TET2 p.G520fs*, TET2 p.Q417*, TET2 p.T229fs*, U2AF1 p.Q157P, ZRSR2 p.R126* |
|  |  |  |  |  |  | 3 | ASXL1 p.R693*, TET2 p.G520fs*, TET2 p.T229fs*, U2AF1 p.Q157P |
| 11 | Male | MF | 66.1 |  |  | 1 | JAK2 p.V617F, ZRSR2 p.R126* |
|  |  |  |  |  |  | 2 | ASXL1 p.Q803*, ASXL1 p.S852*, JAK2 p.V617F, ZRSR2 p.R126* |
| 12 | Male | MF | 72.4 |  |  | 1 | ASXL1 p.R693*, JAK2 p.V617F, PHF6 p.G261R, TET2 p.R544*, ZRSR2 p.K28fs* |
| 13 | Male | ET | 64.3 |  |  | 1 | EZH2 p.C565*, FLT3 p.ITD, IDH2 p.R140Q, JAK2 p.V617F, PHF6 p.R274Q, RUNX1 p.P245fs*, ZRSR2 p.R427fs* |
| 14 | Male | MF | 68.5 |  |  | 1 | ASXL1 p.G629fs*, CALR p.E364fs*, ZRSR2 p.R169* |
| 15 | Male | MF | 68.2 |  |  | 1 | ASXL1 p.Q748*, CALR p.E364fs*, ZRSR2 p.Q114* |
|  |  |  |  |  |  | 2 | ASXL1 p.Q748*, CALR p.L367Tfs*46, IDH1 p.R132H, IDH2 p.R140Q, RIT1 p.M90I, RUNX1 p.I364Gfs*237, SETBP1 p.D868N, SRSF2 p.P95L, ZRSR2 p.Q114* |
| 16 | Male | ET | 49.5 | MF |  | 1 | CALR p.K385fs*, SH2B3 p.D336fs*, ZRSR2 p.P158fs* |
| 17 | Male | MF | 36.3 |  | 64.0 | 1 | MPL p.W515K, PTPN11 p.N308D, RUNX1 p.Q268*, SMC1A p.L571* |
|  |  |  |  |  |  | 2 | ASXL1 p.R693*, ASXL1 p.Y591*, MPL p.W515K |
|  |  |  |  |  |  | 3 | ASXL1 p.R693*, ASXL1 p.Y591*, MPL p.W515K, ZRSR2 p.K98* |
| 18 | Male | ET | 58.2 |  |  | 1 | ASXL1 p.R965*, MPL p.W515L, TET2 p.G1169R, ZRSR2 p.L377fs* |
|  |  |  |  |  |  | 2 | ASXL1 p.R965*, MPL p.W515L, TET2 p.G1169R, ZRSR2 p.L377fs* |
|  |  |  |  |  |  | 3 | ASXL1 p.G629fs*, ASXL1 p.R965*, MPL p.W515L, TET2 p.G1169R, ZRSR2 p.L377fs* |
|  |  |  |  |  |  | 4 | ASXL1 p.R965*, MPL p.W515L, RIT1 p.Q79E, SETBP1 p.D874H, TET2 p.G1169R, ZRSR2 p.L377fs* |
